# Effect of Amino Acid Derivatives and Polyphenol Supplementation on Ovine Muscle Growth In Vitro and In Vivo

**DOI:** 10.3390/muscles5030052

**Published:** 2026-07-17

**Authors:** Gabriella C. Iheanacho, Aliute N. S. Udoka, James L. Klotz, Susan K. Duckett

**Affiliations:** 1 Department of Animal and Veterinary Sciences, Clemson University, Clemson, SC 29634, USA; giheana@g.clemson.edu (G.C.I.); audoka@g.clemson.edu (A.N.S.U.); 2 USDA-ARS, Forage-Animal Production Research Unit, Lexington, KY 40546, USA; james.klotz@ars.usda.gov

**Keywords:** ovine, satellite cells, guanidinoacetic acid, 5-hydroxytryptophan, quercetin, proliferation, differentiation, skeletal muscle growth

## Abstract

Nutrient supplementation may enhance muscle growth by supporting myogenic activity and regulating metabolic pathways involved in skeletal muscle development. However, the effect of specific nutrient supplementation on satellite cell (SC) activity and muscle-related metabolic pathways remains unclear. Two experiments were conducted to investigate dietary supplements, including guanidinoacetic acid (GAA), 5-hydroxytryptophan (5-HTP), and quercetin, on SC proliferation and differentiation in vitro and on muscle growth and transcriptomics in vivo. For the in vitro study, ovine SC were cultured with different levels (0, 12.5, 25, and 50 μmol/L) of 5-HTP, GAA, or quercetin to examine SC proliferation and differentiation. SC proliferation was greater with 5-HTP and quercetin supplementation, whereas GAA increased SC differentiation. Based on these results, 5-HTP and GAA were selected for the in vivo study because they increased proliferation and differentiation, respectively, and had a wider range of dose effectiveness (12.5 and 25 μmol/L). For the in vivo study, Texel lambs (n = 15; 33 ± 6 kg) were blocked by weight and assigned to one of three treatments: control, GAA, or 5-HTP for 28 d. Supplements were administered sublingually at a dose of 2.5 mg/kg body weight daily before feeding. GAA or 5-HTP did not influence (*p* > 0.05) average daily gain or dry matter intake. Longissimus muscle area was greater (*p* < 0.05) at the end of the study compared to the start for all treatment groups. mRNA sequencing showed that GAA supplementation altered gene expression (Padj < 0.05) compared to the control or 5-HTP. However, 5-HTP did not affect (*p* > 0.05) gene expression compared to the control. LC-MS also showed that plasma 5-hydroxyindoleacetic acid (5-HIAA) was elevated (*p* < 0.05) in the 5-HTP group. The results suggest that these dietary supplements can stimulate SC proliferation or differentiation in vitro. Short-term supplementation of GAA or 5-HTP in vivo did not alter growth performance, muscle mass, or miR-133a expression; however, GAA supplementation altered the transcriptome to promote amino acid metabolism pathways that are commonly linked to muscle growth through muscle protein synthesis and energy metabolism.

## 1. Introduction

Satellite cells are muscle progenitor cells, which are responsible for postnatal muscle growth and regeneration. Postnatal muscle growth can occur when there is an increase in muscle fiber size known as hypertrophy. Satellite cells contribute to muscle fiber hypertrophy and regeneration by forming new myonuclei through the process of satellite cell proliferation and differentiation. It has been reported that satellite cells (SCs) constitute about 30–35% of the total number of nuclei along muscle fibers, and the addition of new myonuclei during the postnatal growth stage is essential for myofiber hypertrophy [1,2]. During resistance training, greater SC expansion and myonuclear addition have been linked with more myofiber hypertrophy in humans [3]. The ability to activate SC to enhance proliferation and differentiation has been shown by amino acid derivatives and polyphenol compounds [4,5], but the molecular mechanism of action is still unknown. Many studies report that supplementation of these compounds alters microRNA (miRNA) expression. Some miRNAs are labeled as myomiRs due to their predominant expression in skeletal muscle [6]. miRNAs play a significant role in muscle growth by regulating gene expression at the post-transcriptional level [7]. miR-133a is regarded as a myomiR, meaning that miR-133a is predominantly expressed in muscle, where it regulates myogenic differentiation [8]. Wang et al. [9] reported that GAA supplementation caused downregulation of miR-133a-3p for the C2C12 cell line, leading to an increase in myotube growth. Human studies have also shown that resistance exercise training-induced skeletal muscle hypertrophy is associated with changes in miRNA expression, suggesting that miRNAs contribute to individual variations in muscle growth responses [10].

Many dietary supplements on the market for humans are shown to alter muscle growth in vitro and in vivo [4]. Amino acid derivatives and polyphenols are naturally occurring compounds that have been investigated to improve muscle growth and regeneration in humans [5]. Several studies have investigated the effect of nutrient supplementation on satellite cells in animals and highlighted its effect on muscle growth [11,12,13,14,15,16]. Based on this, we chose to examine guanidinoacetic acid (GAA), 5-hydroxytryptophan (5-HTP), and quercetin to enhance ovine muscle growth due to their ability to stimulate creatine and energy metabolism [17], serotonin-induced smooth muscle hyperplasia and hypertrophy [18], or reduce inflammatory muscle fiber atrophy [19], respectively. Quercetin, a polyphenol that can be obtained from fruits and vegetables, has been shown to increase myoblast differentiation [20], and it plays a vital role in skeletal muscle growth by influencing satellite cell activities that could lead to muscle hypertrophy, reducing muscle atrophy, and promoting muscle regeneration [21]. Similarly, GAA, an amino acid derivative, has been reported to promote muscle development and increase the body’s energy reserve by increasing the creatine and adenosine triphosphate load in the body, thereby enhancing growth performance [22,23,24]. GAA supplementation in broiler diets has also been shown to improve breast meat yield [25], and it may influence muscle development by regulating the expression of numerous genes in the MAPK signaling pathway through miR-1a-3p, miR-130b-5p [26]. 5-HTP is a precursor to serotonin and an amino acid derivative that influenced the performance of calves, resulting in a higher average daily gain [27]. There are no growth-enhancing compounds approved for use in sheep (ruminants) in the United States that could be used to stimulate muscle growth. Exploration of natural supplements that enhance muscle growth in sheep would be advantageous to improve lamb meat yield and enlarge lamb chop size. Evidence shows that GAA is degraded in the rumen of cattle, with only 50% bypassing ruminal degradation when infused directly into the rumen [28] and ruminal degradation of 5-HTP is unknown; therefore, we used a sublingual delivery method for these compounds in lambs to bypass potential rumen degradation. Despite growing interest in the relationship between skeletal muscle growth and nutrient supplementation, limited information is available on how GAA, 5-HTP, and quercetin influence satellite cell function and muscle-related molecular pathways in controlled animal experiments. A better understanding of these responses may provide insight into how targeted nutritional strategies regulate myogenesis, metabolism, and muscle development. We hypothesize that quercetin, GAA, or 5-HTP would alter ovine satellite cell proliferation and/or differentiation, miR-133a expression, and the transcriptome to promote ovine skeletal muscle growth in vitro and in vivo. The objectives of this study were to: (1) investigate the effect of quercetin, GAA, and 5-HTP on ovine satellite cell proliferation and differentiation in vitro, and select the two best supplements for an in vivo experiment; and (2) examine the effect of GAA and 5-HTP on ovine miR-133a expression, transcriptomics, and muscle growth in vivo.

## 2. Results

**Figure 1:**
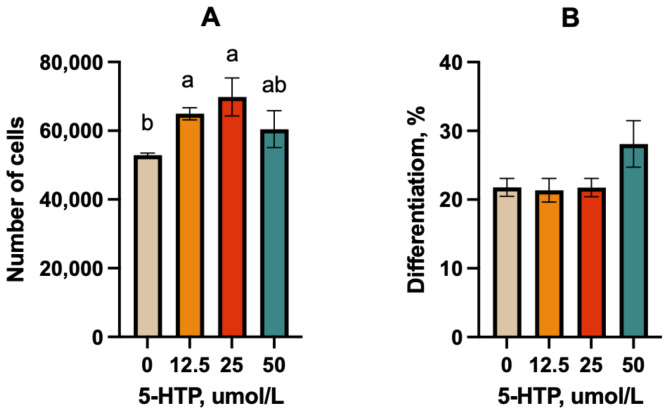
Effect of 5-hydroxytryptophan (5-HTP) addition to ovine primary satellite cells (SC) in vitro on: (**A**) number of EDU-stained proliferating SC, and (**B**) percentage of SC that differentiated (MYOG+). Each color bar represents a treatment concentration, and bars represent mean ± standard error of the mean. ^ab^ Letters with uncommon superscripts differ (*p* < 0.05).

***Experiment 1.*** Satellite cell (SC) proliferation was affected by 5-HTP addition (*p* < 0.05). A cell proliferation kit using 5-ethynyl-2′-deoxyuridine (EDU), which incorporates into newly synthesized DNA with a fluorescent tag for imaging (red), showed that 5-HTP at 12.5 and 25 μmol/L had a greater (*p* < 0.05) number of proliferating SC than 0 μmol/L (Figure 1A). SC differentiation was not affected (*p* > 0.05) by 5-HTP addition at any concentration (Figure 1B).

**Figure 2:**
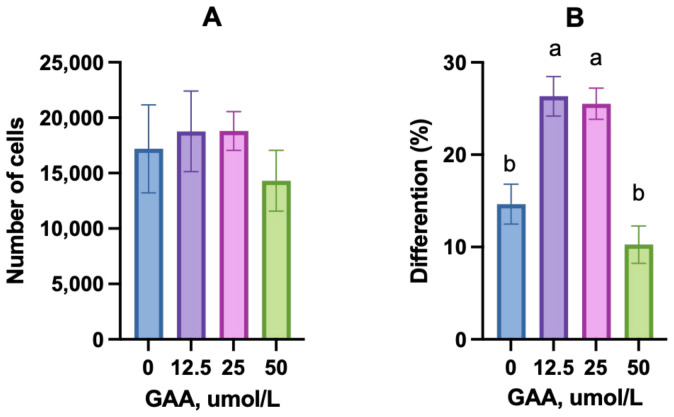
Effect of guanidinoacetic acid (GAA) addition to ovine primary satellite cells (SC) in vitro on: (**A**) number of EDU-stained proliferating SC, and (**B**) percentage of SC that differentiated (MYOG+). Each color bar represents a treatment concentration, and bars represent the mean ± standard error of the mean. ^ab^ Letters with uncommon superscripts differ (*p* < 0.05).

GAA addition at any concentration did not alter (*p* > 0.05) SC proliferation compared with 0 μmol/L (Figure 2A). GAA increased SC differentiation (*p* < 0.05), with 12.5 μmol/L and 25 μmol/L having the highest percentage of differentiating cells when compared with 0 μmol/L (Figure 2B). Others have reported that GAA alters miR-133a-3p expression [9] in C2C12 cell lines, so we examined this in our GAA in vitro experiment. miR-133a-5p and miR-133a-3p relative expressions using U6 as the reference gene were measured in the GAA in vitro treatments (n = 2/concentration) at the end of d 4. For miRNA gene expression in vitro, miR-133a-5p yielded an undetermined Ct (cycle threshold) value, indicating that the target was not detected within the amplification range. However, miR-133a-3p was in low abundance during proliferation and showed no difference (*p* > 0.05) between GAA treatment concentrations during differentiation compared to the control.

**Figure 3:**
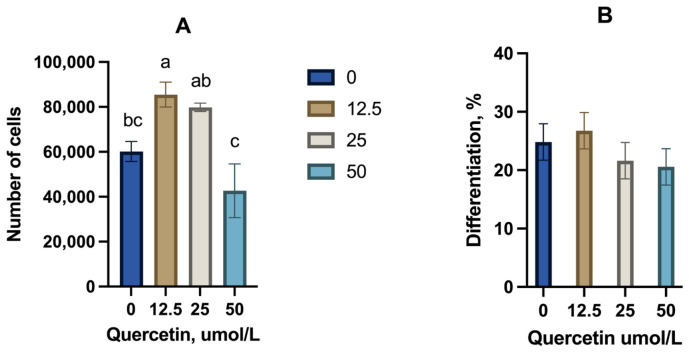
Effect of quercetin addition to ovine primary satellite cells in vitro on: (**A**) number of EDU-stained proliferating SC, and (**B**) percentage of SC that differentiated (MYOG+). Each color bar represents a treatment concentration, and bars represent mean ± standard error of the mean. ^abc^ Letters with uncommon superscripts differ (*p* < 0.05).

Quercetin addition at 12.5 μmol/L increased (*p* < 0.05) the number of proliferating SC compared to 0 or 50 μmol/L quercetin (Figure 3A). Quercetin addition, at any concentration, did not influence satellite cell differentiation (*p* > 0.05) compared to 0 μmol/L (Figure 3B).

***Experiment 2.*** Based on the results from the in vitro experiment, 5-HTP and GAA were selected to use in the in vivo study. 5-HTP was selected because it increased SC proliferation and was effective at a wider range of doses than quercetin. GAA was selected based on increased SC differentiation. It is unknown which stimulus, increased proliferation or increased differentiation from the in vitro SC results, would translate into greater impact on molecular mechanisms underlying skeletal muscle hypertrophy in finishing lambs.

The LC-MS analysis for plasma 5-Hydroxytryptamine (5-HT), 5-Hydroxytryptophan (5-HTP), 5-Hydroxyindoleacetic acid (5-HIAA), acetate (AC), hydroxybutyrate (HB), propionate (Prop), isobutyrate (Isob), isovalerate + 2-Methylbutyrate (IVMB), hippurate (Hipp), succinate (Succ), and guanidinoacetic acid (GAA) are shown in Table 1. 5-HIAA was greater (*p* < 0.05) for the 5-HTP-supplemented group compared with the control and GAA groups. Concentrations of other serotonin metabolites, short-chain fatty acids or GAA did not differ by treatment.

**Figure 4:**
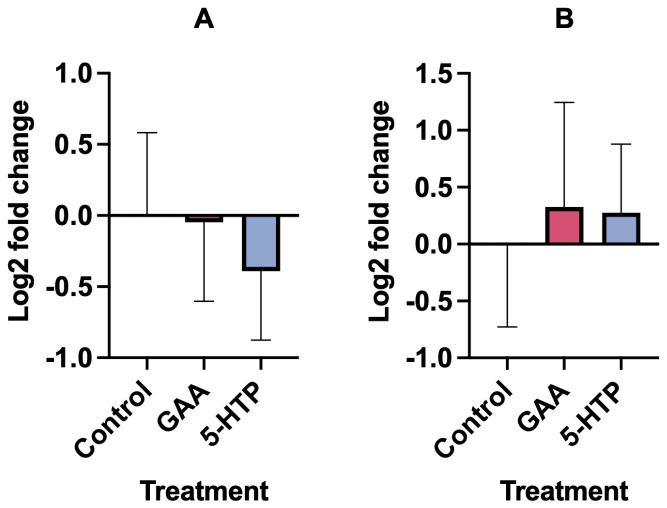
Expression of miR-133a-3p (**A**) and miR-133a-5p (**B**) for different treatment groups: control (CON), guanidinoacetic acid (GAA) or 5-hydroxytryptophan (5-HTP). The x-axis represents the treatment groups, and the y-axis represents relative miRNA expression expressed as log2 fold change. Expression was normalized using U6 and expressed relative to the control group using the 2^−ΔΔCT^ method. Bars represent mean ± standard error of the mean.

In vivo treatment effect of GAA and 5-HTP on miRNA expression in longissimus muscle biopsies showed that 5-HTP or GAA did not affect (*p* >0.05) the expression of miR-133a-3p (Figure 4A) and miR 133a-5p (Figure 4B) compared with the control.

Following mRNA sequencing, total raw reads generated per individual sample ranged from 73,462,595 to a minimum of 50,000,000, and all samples had Q30 > 97%. After removing and filtering reads containing poly A/T/G/C, sequences containing >10% (<0.0001%; almost no reads had more than 10% unknown bases) N content, low-quality sequences, and those with 5′ splices lacking 3′ splices or insertions, the remaining reads were used for mapping. The result of filtering (Table S1) showed that all sequenced samples were suitable for further data processing.

**Figure 5:**
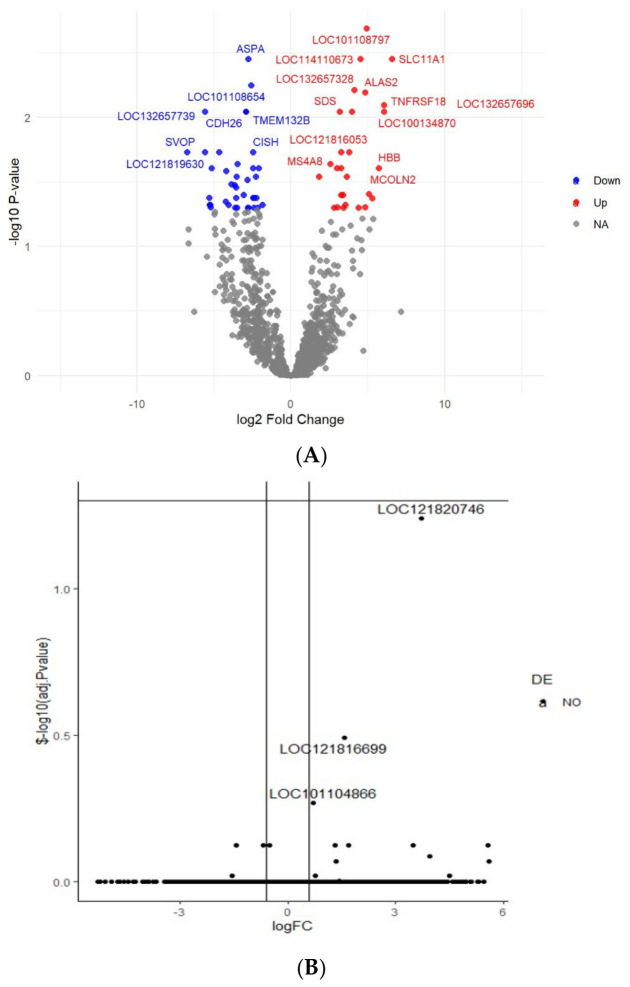

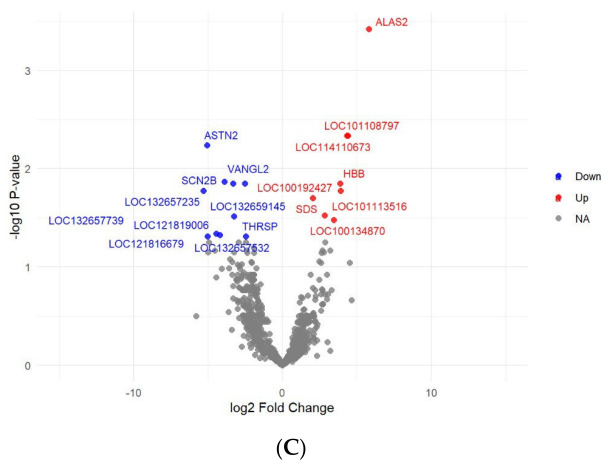
Volcano plot showing differentially expressed genes with fold change and statistical difference for the guanidinoacetic acid (GAA) vs. control (Con) treatment group (**A**), 5-hydroxytryptophan (5-HTP) vs. control treatment group (**B**), and the GAA vs. 5-HTP treatment group (**C**) (LogFC > 1; adj *p* value < 0.05). Each point represents an individual gene plotted by log2 fold change on the x-axis and −log_10_ on *p*-value on the y-axis. Red points indicate upregulated genes, blue points indicate downregulated genes, and gray points represent genes that are not differentially expressed.

GAA supplementation resulted in differentially expressed genes (adj *p* < 0.05) compared to the control (Figure 5A). GAA upregulated 27 genes and downregulated 38 genes compared to the control. In contrast, there were no differentially expressed genes (adj *p* > 0.05) in 5-HTP-supplemented muscle compared to controls (Figure 5B). For the GAA vs. 5-HTP comparison, GAA upregulated (adj *p* < 0.05) eight genes and downregulated 10 genes (Figure 5C) compared to 5-HTP. Upregulated genes in GAA vs. Con and GAA vs. 5-HTP include MYC, ALAS2, and MS4A8, while genes that were downregulated include ENOX2 and ASTN2.

**Figure 6:**
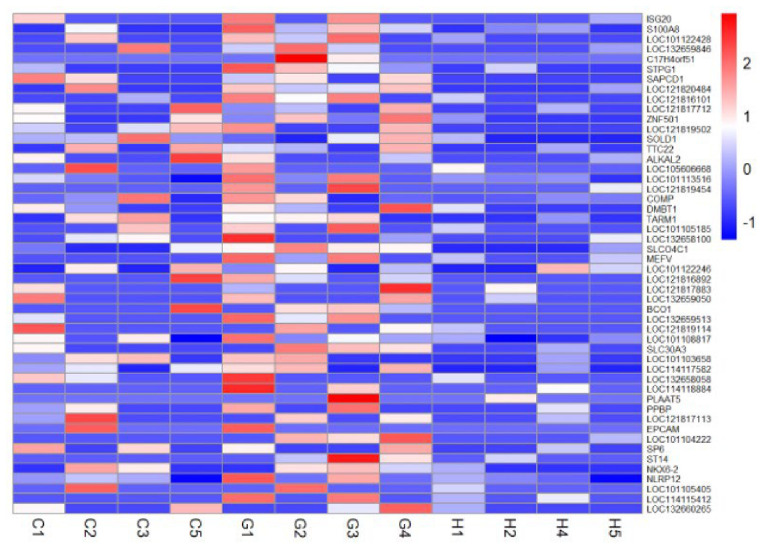
Heatmap of the DE genes with DESeq normalized data (C = Control, G = guanidinoacetic acid (GAA), and H = 5-hydroxytryptophan (5-HTP)). Rows represent individual genes, and columns represent biological samples. Gene expression values are scaled related to expression, with red indicating higher expression and blue indicating lower expression.

The heatmap for gene expression across all samples is shown in Figure 6. Expression patterns of selected genes across control (C), 5-HTP (H), and GAA (G) samples are displayed. Compared with the control, GAA-treated samples showed increased expression of several genes, whereas 5-HTP showed a mixed expression pattern, indicating a different response to GAA. Upregulated genes in GAA vs. Con and GAA vs. 5-HTP enriched eight pathways (adj *p* < 0.05; Table 2). Smaller adjusted FDR values indicate stronger evidence that the pathway is enriched. nGenes denotes the number of differentially expressed genes that match the pathway while pathway gene shows the total number of genes annotated in each pathway.

**Figure 7:**
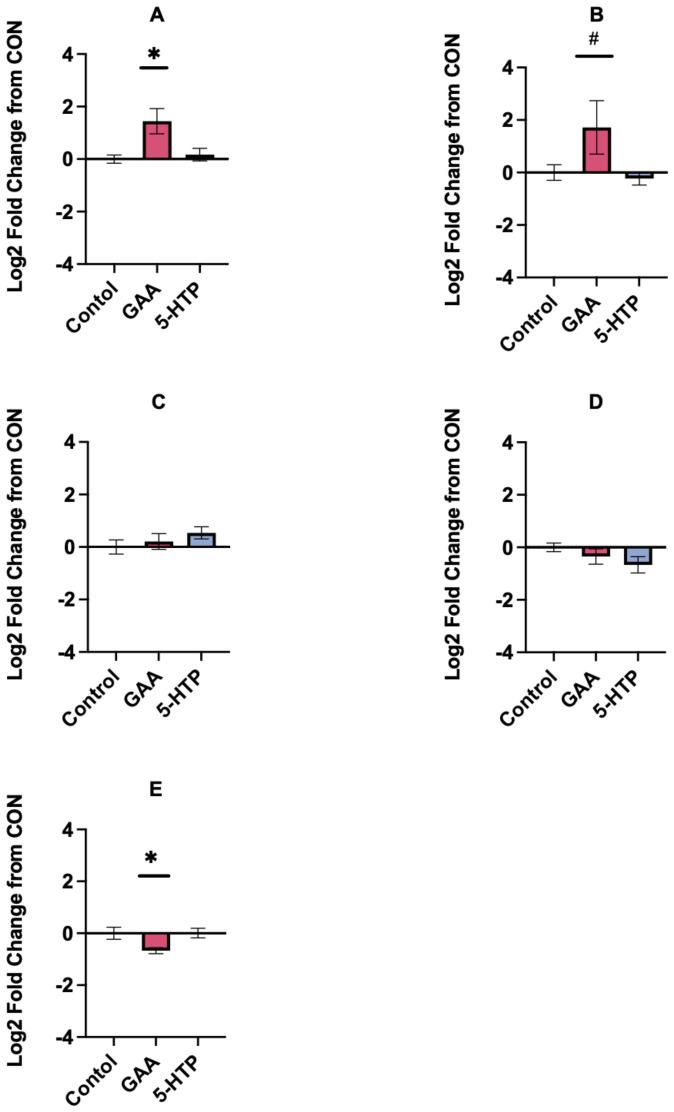
Quantitative PCR (qPCR) validation of gene expression for selected differentially expressed genes (MYC (**A**), MS4A8 (**B**), MYOD (**C**), PAX7 (**D**) and ENOX2 (**E**)) identified from DESeq2 analysis in longissimus muscle samples from lambs assigned control (CON), guanidinoacetic acid (GAA) or 5-hydroxytryptophan (5-HTP). The x-axis represents the treatment groups, and the y-axis represents relative mRNA expression expressed as log2 fold change. Gene expression was normalized using reference genes EIF3K and UXT and presented as log2 fold change from CON. * (*p* < 0.05) # (*p* < 0.10).

qPCR validation of RNA-seq DEGs (Figure 7) showed that, relative to the control, GAA increased MYC (*p* < 0.05) and MS4A8 (*p* < 0.10) expression and decreased ENOX2 (all *p* < 0.05), whereas MYOD and PAX7 did not differ between treatments (*p* > 0.05). 5-HTP did not show any difference (*p* > 0.05) in gene expression when compared with the control. These results agree with the sequencing results in this study.

**Figure 8:**
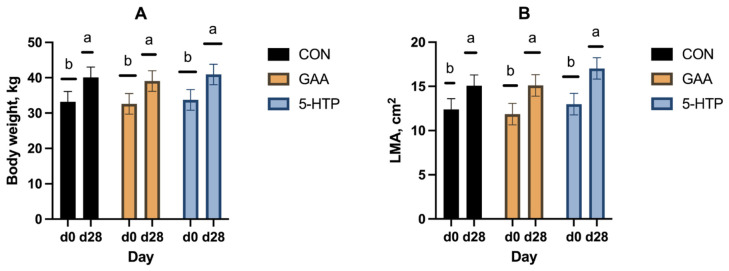
Body weight (BW, n = 5/treatment) (**A**) and ultrasound longissimus muscle area (LMA, n = 5/treatment) (**B**) at 12th/13th rib before the study started (d0) and at the end of the 28 d supplementation period (d28) for lambs supplemented with control (CON), guanidinoacetic acid (GAA; 2.5 mg/kg BW) or 5-hydroxytryptophan (5-HTP; 2.5 mg/kg BW). ^ab^ Means with uncommon superscripts differ by day (*p* < 0.05). Treatment and the interaction with day were non-significant.

Lamb body weight increased (*p* < 0.05) from d 0 to d 28; however, there were no differences between treatments (Figure 8). Lamb longissimus muscle area increased (*p* < 0.05) from d 0 to d 28, but changes were similar between treatments.

## 3. Discussion

Satellite cells (SC) are muscle progenitor cells that play an important role in postnatal muscle growth and regeneration. 5-HTP supplementation in vitro stimulated SC proliferation at 12.5 and 25 μmol concentrations; however, SC differentiation was not affected. 5-HTP is a direct precursor to serotonin [29]. Serotonin has been shown to regulate smooth muscle stem cell (SMC) growth in vitro through dual mechanisms: an intracellular growth-stimulatory effect and inhibitory effects due to cell-surface action [30]. GAA supplementation increased satellite cell differentiation in vitro but did not affect satellite cell proliferation. GAA is a direct precursor to creatine, and creatine monohydrate has been reported to increase satellite cell differentiation but did not increase proliferation of satellite cells over the control culture [31]. Similarly, Dodson et al. [32] reviewed factors that regulated satellite cell activities, and they found that creatine showed a steady effect on satellite cell differentiation in vitro. Wang et al. reported that GAA reduced the proliferation of C2C12 cells, but it promoted myoblast differentiation through miR-133a-3p and miR-1a-3p by causing activation of the Akt/mTOR/S6K signaling pathway [9]. In this study, miR-133a-3p expression was not altered in vitro with 5-HTP or GAA supplementation and miR-133a-5p was not expressed. Quercetin supplementation in vitro altered SC proliferation without altering SC differentiation in this study. In contrast, others have shown that quercetin increases myoblast fusion and myogenic differentiation in mouse C2C12 cells [20]. These results demonstrate that each supplement tested has different effects on SC proliferation or differentiation at specific concentrations tested. Overall, 5-HTP and quercetin stimulated SC proliferation, whereas GAA stimulated SC differentiation. We chose to examine 5-HTP and GAA in a subsequent in vivo study due to the differing effects on SC proliferation versus differentiation to determine which may have greater effects on muscle growth in vivo.

5-Hydroxyindoleacetic acid (5-HIAA) is the main breakdown product of serotonin. In this study, plasma 5-HIAA content was elevated in lambs that received a 5-HTP supplement through the sublingual route. This shows that there was a breakdown of 5-HTP after supplementation, and the conversion of serotonin to 5-HIAA can be prompted by the metabolites in the gut microbiome [33,34]. Our current finding suggests that 5-HTP was effectively metabolized through the serotonin pathway, which led to an increase and subsequent catabolism to 5-HIAA. Since the effect was unique to the 5-HTP supplementation group, it shows that 5-HTP is modulating serotonin metabolism. The elevation of 5-HIAA may reflect an increase in the utilization of serotonin that supports anabolic processes or adaptive responses in skeletal muscle, since serotonin has been reported to promote the growth of longitudinal muscle fiber in transgenic mice in vitro [35]. Rumen-protected 5-HTP fed to 3-year-old Kazakh female sheep for 25 days has shown an increase in 5-HIAA content in blood plasma [36]. Plasma GAA concentration was not altered in 5-HTP or GAA-supplemented lambs. In dogs, sublingual delivery of Biphenol A resulted in rapid absorption into the bloodstream with a peak at 10–13 min depending on dose [37]. In our study, we collected blood samples prior to administering the sublingual dose, and therefore, the lack of change in 5-HTP or GAA in plasma samples observed may be related to the sampling time and indicates that the sublingual delivery does not elevate the compound of interest for a sustained time in the circulation. This would indicate that more frequent delivery of compounds via the sublingual route would be needed to maintain elevation of these compounds in circulation.

GAA supplementation altered the transcriptome compared to control or 5-HTP treatments, and its enriched pathways are shown in Table 2. According to the pathway enrichment analysis, glycine–serine–threonine metabolism and cysteine–methionine metabolism are the amino acid metabolism pathways with the highest number of pathway genes, indicating that GAA affects amino acid utilization and downstream energy metabolism, which are functions characteristic of a precursor to creatine. The biosynthesis of valine, leucine, and isoleucine, which are branched-chain amino acids, is said to be useful in skeletal muscle because they can be broken down and used as a source of energy [38]. GAA is produced from glycine and arginine, then undergoes methylation to form creatine using S-adenosylmethionine (SAM), which is derived from methionine. This suggests that GAA supplementation may alter methyl-group metabolism and increase creatine availability in livestock, especially when methionine is scarce. Ringel et al. found that GAA supplementation increased creatine content in broiler breast muscle, suggesting that GAA can be used as a good source of creatine [39]. Similarly, Asiriwardhana & Bertolo reported a possible increase in creatine production when GAA supplementation elevates methyl group consumption from SAM [40]. These results suggest that GAA may be altering protein synthesis/degradation based on enrichment of genes in amino acid utilization and energy metabolism. Research in cattle with GAA supplementation has shown that responses are tissue-specific, with skeletal muscle altering genes regulating intramuscular fat deposition in their study [41]. However, in this study, RNA sequencing did not identify any lipogenic genes with altered transcription levels.

5-HTP supplementation did not alter gene expression compared to the control. Serotonin, derived from 5-HTP, has been reported to modulate physiological functions such as appetite [42] and may not have influenced gene expression at the transcriptomic level. Despite the known metabolic influence of 5-HTP, our findings showed no gene expression changes in skeletal muscle compared to the control. However, there were differences in gene expression between 5-HTP and GAA. The lack of differentially expressed genes between 5-HTP and control may be attributed to the period of supplementation, transitional effects, or regulatory mechanisms that bypass mRNA-level transcriptional changes. Others have reported that 5-HTP supplementation at higher doses (90 mg/d) for 10 days postweaning (18 d of age) can alter the expression of several genes involved in muscle and adipose tissue growth [43]. It is important to note that at this age (18 d), calves would be pre-ruminants without a fully developed rumen, and therefore, supplementation via milk replacer would allow the 5-HTP to bypass into the abomasum for direct absorption [43]. They found that 5-HTP downregulated immune-related pathways, cell cycle pathways, and histone modifications. At the microstructural level, they did not find any change in the muscle fiber cross-sectional area but did report smaller adipocyte cross-sectional areas with 5-HTP supplementation. 5-HTP may have shifted its metabolic effect without producing strong, detectable transcriptional effects in the tissue since 5-HTP is a precursor to serotonin and 5-HIAA is the byproduct of serotonin breakdown [44]. qPCR analysis of selected genes did confirm our sequencing results in that GAA upregulated MYC and MS4A8 without altering PAX7 or MYOD. GAA downregulated ENOX2, which is involved in cell growth regulation; however, most is known about ENOX2 in cancer cell development, where it shows upregulation associated with greater tumor growth [45]. MYC is known to be a transcription factor that regulates cellular plasticity [46,47,48], and its role in muscle fiber growth has drawn research attention over the years [49,50]. Murach et al. [46] reported that during the early phase of muscle fiber growth, MYC is involved in various aspects of gene expression, and our current study indicated that GAA upregulated MYC compared to the control or 5-HTP group. This shows that GAA is a potential promoter for skeletal muscle growth through MYC-related mechanisms. Von Walden et al. [51] reported that MYC protein is associated with the ribosomal DNA during muscle loading and influences muscle hypertrophy. Similarly, the findings of Chaillou et al. [52] highlighted that MYC, as a transcription factor, can enhance ribosomal RNA transcription, an important step in protein synthesis and biogenesis during muscle hypertrophy. SHISA5 is known as an endoplasmic reticulum-associated protein [53]. Given that insulin-like growth factor-1 (IGF-1) can be modulated by endoplasmic reticulum stress response [54], upregulation of SHISA5 in response to GAA supplementation may influence IGF-1 signaling pathways. This interaction could contribute to long-term muscle hypertrophy by coordinating anabolic signaling with cellular homeostasis during muscle remodeling.

Our study showed that GAA or 5-HTP supplementation did not influence miR-133a-3p expression in skeletal muscle. A similar outcome was observed when we analyzed the effect of GAA or 5-HTP on miRNA 133a-5p. Others have shown that GAA supplementation has been shown to promote skeletal muscle growth by downregulation of miR-133a-3p and miR-1a-3p in C2C12 cells [9]. Greene et al. discovered that muscle fiber increases in cross-sectional area along with changes in miRNA expression from the prenatal to postnatal stage of muscle growth [55]. They reported that miR-133a expression was upregulated in the skeletal muscle of lambs during prenatal and early postnatal skeletal muscle development, indicating that it may have a role in skeletal muscle hypertrophy, which occurs during this time period.

Lamb performance and longissimus muscle area as measured by ultrasound were not altered in this study with sublingual supplementation of GAA or 5-HTP. This lack of effect on growth and muscle mass in this study may be related to the small number of animals per treatment used in this study. Others have also shown that GAA supplementation did not show any effect on feed-to-gain, average daily gain (ADG), and average daily feed intake of growing finishing pigs [56]. In contrast, Jin et al. reported that 90 days of supplementation with GAA improved different muscle parameters, including ADG, fiber diameter, and the shear force of the Hu sheep (BW= 16.91 ± 3.1 kg) [57]. In cattle, GAA supplementation at 0.6 and 0.9 mg/kg dry matter improved intake, average daily gain, and feed efficiency via altering rumen fermentation [41,58,59]. Others have shown that 5-HTP supplementation improves performance of calves [27] and skeletal muscle transcriptomics [43]. Our results could be related to the short-term supplementation and/or the delivery of the supplement, which could impact efficacy. Extrapolation of in vitro treatment concentrations that promoted SC proliferation or differentiation to in vivo dosing is challenging, and therefore, we used dose levels that had already been reported in the literature [43,57]. The use of the longissimus muscle biopsy also limits the amount of sample for downstream analyses. This study focused on molecular changes in the skeletal muscle related to GAA and 5-HTP in vivo supplementation, and the limited sample amount with biopsy did not allow for SC isolations or muscle fiber cross-sectional area measurements, which would have helped to relate potential modes of action from both the in vitro and in vivo research.

## 4. Materials and Methods

### 4.1. Experiment 1

***Satellite Cells***. Satellite cells used for this study were previously isolated from the longissimus thoracis et lumborum muscle (LM) of 2-day-old lambs [60]. All animal and experimental protocols were approved by the Clemson University Institutional Animal Care and Use Committee (AUP2019-0078). Individual technical replicate experiments (n = 3/concentration/supplement) were conducted using the same SC population to examine each supplement at four different concentrations. For proliferation and differentiation assays, satellite cells per well were plated at a starting concentration of 45,000/well in 24-well plates (Corning, Corning, NY, USA, ThermoFisher, Waltham, MA, USA) with two wells per treatment concentration. Plates were coated with fibronectin (10 μg Fb/mL, Millipore Sigma) according to Dodson et al. [61]. Fibronectin (500 µL) was added into each well and allowed to sit for 1 h at room temperature in a laminar flow hood, after which it was removed using a serological pipette, and the culture media was added afterward.

***Cell proliferation assay.*** Satellite cells (45,000/well) were plated in a proliferation medium comprising 20% fetal bovine serum (FBS), 1% antibiotics/antimycotics, 0.1% gentamycin, and DMEM (Dulbecco’s Modified Eagle Medium). The media contained either guanidinoacetic acid (GAA; Sigma G11608, St. Louis, MO, USA), 5-hydroxytryptophan (5-HTP; Bulk Supplements, Henderson, NV, USA), or quercetin (Bulk Supplements) at a concentration of 0 (control), 12.5, 25, and 50 µmol/L. Supplements were added to basal media and run as individual experiments, with each concentration run in duplicate. After plating, the cells were incubated at 37.0 °C with 5.0% CO_2_ (ThermoFisher Incubator). The media was changed every two days.

Beginning at 24 h (d 1) after plating, cells were fixed with 1 mL of 4% formaldehyde in PBS (phosphate-buffered saline) following staining to assess differences in proliferation. A single plate for each treatment was brought out from the incubator, viewed under the microscope to ensure no contamination, and then transferred into the laminar flow hood. The media was removed using a serological pipette, and the cells were fixed by adding 1 mL of 4% formaldehyde in PBS into each well and incubated for 15 min at room temperature. The fixative was removed after 15 min, and each well was washed once with 1 mL of PBS. Once the wash solution was removed, 1 mL of Hoechst 33,342 (10 μg/mL, ThermoFisher) in a 1:2000 dilution was added to each well and incubated for 30 min at room temperature (protected from light). After 30 min, the Hoechst dye was removed, and each well was washed twice with 1 mL of PBS, followed by imaging using a BioTek Cytation imaging reader (Winooski, VT, USA), and nuclei that fluoresce with a blue color were captured to obtain a cell count.

At 96 h (d 4) for proliferation staining, cells were allowed to reach 70–80% confluency before 5-ethynyl-2-deoxyuridine (EDU) staining was carried out. 5-ethynyl-2-deoxyuridine (Edu) staining was used to visualize the proliferation of the satellite cells on day four of culture using the ClickIT Edu Alexa Fluor 555 (ThermoFisher) Cell Proliferation Assay Kit according to the manufacturer’s protocol, and cells were incubated at 37.0 °C with 5.0% CO_2_ (ThermoFisher Incubator) to allow the EdU to be incorporated in actively proliferating cells. The BioTek Cytation imaging reader was used to visualize the proliferating cells that fluoresce in both blue and red. Images were exported to an Excel file and automatically counted using ImageJ (https://imagej.net/ij/ (accessed on 7 July 2025)). The total number of proliferating SC was counted using EDU-stained cells. Counts were recorded for further statistical analysis. Representative images of EDU-stained cells for each treatment and concentration are shown in Figure S1.

***Cell differentiation and immunocytochemistry staining.*** To estimate differentiation capacity, satellite cells were plated at 45,000 cells per well. When the cells reached 70–80% confluency in a proliferation medium, differentiation was induced by changing the media to a differentiation medium comprising 2% FBS, 1% antibiotics/antimycotics, 1% gentamycin, and DMEM (Dulbecco’s Modified Eagle Medium). The media contained either guanidinoacetic acid (GAA), 5-hydroxytryptophan (5-HTP), or quercetin at an inclusion level of 0 (control), 12.5, 25, and 50 μmol/L. This was followed by four days of incubation, and the media was changed every two days.

Immunocytochemistry staining was carried out on day four, following Danoviz & Yablonka [62]. In brief, cells were fixed with 4% paraformaldehyde in PBS and allowed to sit for 10 min at room temperature. Triton X-100 (500 µL of 0.5%) in Tris-buffered saline (TBS) was added to each well, followed by blocking solution (500 µL) of 1% normal goat serum (NGS) in TBS. Plates were kept at 4 °C overnight. The primary antibody staining started by allowing the plate to warm up at room temperature and diluting the primary antibody for MyoG (F5D; DSHB, Iowa City, IA, USA) to 0.05 μg/μL. Primary antibody (150 µL; 0.05 µg/µL) was added into each well, followed by 1 h of incubation at room temperature, after which it was transferred to a 4 °C refrigerated chamber with a light and continuous swirling on a flat surface overnight. The plate was allowed to warm at room temperature, and the secondary antibody was diluted (Alexa Fluor 488 goat anti-mouse IgG1; 1:2000 dilution; ThermoFisher, Waltham, MA, USA). The culture was rinsed 3 times using 0.05% Tween-20 in TBS, after which 150 µL of the diluted secondary antibody was added into each well and allowed to sit at room temperature for 2 h in the same swirling and flat surface conditions. The secondary antibody was removed, and each well was washed 3 times using 0.05% Tween-20 in TBS. For nuclear visualization, 100 µL of Hoechst solution (5 µg/mL) diluted in TBS was added to each well and allowed to incubate for 30 min at room temperature. The culture was rinsed twice with 0.05% Tween-20 in TBS and a final rinse with TBS. Vectashield (Newark, CA, USA) mounting medium (3 µL) was added to each well, followed by 25% glycerol in PBS. The plate was imaged using a BioTek Cytation imaging reader for cells that fluoresce in blue and green. The percentage of differentiating cells was obtained by using ImageJ to count the total number of cells and the cells expressing myogenin (MYOG). Representative images of MYOG-stained cells for each treatment and concentration are shown in Figure S2.

***RNA and miRNA.*** RNA was harvested on day four of culture using Trizol (Invitrogen, Thermo Fisher Scientific, Waltham, MA, USA). A plate was brought from the incubator, viewed under the microscope, and transferred to the laminar flow hood. Media was removed using a serological pipette, after which 125 µL of Trizol reagent was added into each well. The plate was rocked gently to ensure the reagent was dispersed adequately around the bottom of each well and allowed to sit for 5 min. After 5 min, the Trizol from the different concentrations of each supplement was transferred into a 1.7 mL tube and stored at −80 °C for subsequent RNA extraction. RNA was extracted according to the Trizol procedure. We quantified the extracted RNA using a NanoDrop 1 spectrophotometer (Thermo Scientific, ThermoFisher, Waltham, MA, USA).

According to the manufacturer’s instructions, miRNA cDNA was obtained from the isolated RNA using the TaqMan miRNA RT Kit (ThermoFisher, Walthan, MA, USA) for miRNA evaluation. Quantitative real-time RT-qPCR (qPCR) using the TaqMan Fast Advanced Master Mix (ThermoFisher, Walthan, MA, USA) and the QuantStudio 3 (ThermoFisher, Walthan, MA, USA) was used to analyze miR-133a-3p and miR-133a-5p (ThermoFisher TaqMan MicroRNA Assays). U6 (ThermoFisher TaqMan MicroRNA Assays) was used as a reference gene to normalize the expression levels of the target gene across the different samples.

***Statistical analysis.*** Satellite cell proliferation and differentiation for each treatment (GAA, 5–HTP, or Quercetin) at four concentrations (0, 12.5, 25, and 50 μmol/L) were analyzed using Analysis of Variance (ANOVA) using JMP PRO 17.1.0 (971353). The statistical model included treatment. Significance was determined at *p* < 0.05, and the difference between treatment concentrations was assessed using the LSMean paired Student’s *t*-test. Power was adequate (*p* = 0.90) with three technical replicates per concentration of each treatment to detect a significant difference (α = 0.05) given average response and standard deviations from previous primary SC experiments on proliferation and differentiation [55].

### 4.2. Experiment 2

***Experimental design.*** The use of animals for this experiment was approved by the Clemson University Institutional Animal Care and Utilization Committee (AUP2024-0217). Texel lambs (n = 15; 33 kg ± 6 kg BW; 7 mo of age) were used in this experiment. The experiment was conducted at the Clemson University Small Ruminant Facilities, Lebanon Rd, Pendleton, USA, and lasted 28 days. The lambs (3 male and 2 female/treatment) were randomly assigned to one of three treatments: control (diluent only), 5-HTP (2.5 mg/kg BW), and GAA (2.5 mg/kg BW). Feed (75% Purina Lamb Grower and 25% whole corn; 12.5% crude protein, Arden Hills, MN, USA) was provided ad libitum individually. Lambs had free access to water and trace mineral supplementation. Dose concentrations of supplements were similar to those reported by others for 5-HTP and GAA in vivo [43,57]. Treatments were administered individually to lambs via a sublingual dose prior to feeding each day. The treatments were dissolved in 200 μL of water first and vortexed for 10 s, followed by the addition of 300 μL of PBS, 300 μL of DMSO (dimethyl sulfoxide), and 200 μL of PEG (polyethylene glycol) sequentially, and vortexed to mix properly [63,64]. Control lambs received only the diluent used to dissolve the other treatments. Lamb weights were taken on two consecutive days at the beginning and end of the study. Feed intake and refusals were recorded for each lamb daily.

***Real-time ultrasound.*** Longissimus muscle area (LMA) was measured at the start and end of the experiment using real-time ultrasound technology. The ultrasound scan was performed on the longissimus muscle between the 12th and 13th ribs using an Aloka 500 V ultrasound (Corometric Medical Systems, Wallingford, CT, USA) equipped with a 17 cm, 3.5 MHz linear probe. The images were interpreted by Biosoft Toolbox (Biotronic, Inc., Ames, IA, USA). The outline of the ribeye muscle was traced and recorded for further analysis. Lamb body weight was also recorded for two consecutive days and averaged at the start and end of the study.

***Blood samples.*** Blood samples were collected at the end of the experiment from the jugular vein of the lambs using a 10 mL Vacutainer blood collection tube containing EDTA (BD Vacutainer, Becton, Dickinson and Company, Franklin Lakes, NJ, USA); a Vacutainer needle holder; and an 18-gauge, 3.81 cm multi-sample Vacutainer needle (VWR, Batavia, IL, USA). After blood collection, the blood collection tube was inverted and immediately placed on ice for further processing. Blood samples were centrifuged at 2000× *g* for 20 min at 4.0 °C. Plasma was then collected and aliquoted into 1.7 mL tubes with 1 mL of plasma per tube. Samples were stored at −80 °C until subsequent analyses.

***Plasma Metabolites.*** Plasma (100 μL) underwent protein precipitation via the addition of acetonitrile containing eight isotopically labeled internal standards (including guanidinoacetic acid-d2 (CDN Isotopes Inc., Vaughan, ON, Canada)). Samples were vortexed and centrifuged. Derivatizing reagents (3-nitrophenylhydrazine (3-NPH) and 1-ethyl-3-(3-dimethylaminopropyl) carbodiimide (EDC) containing 7% pyridine) were added (and samples heated) to create 3-NPH derivatives of guanidinoacetic acid and other VFA’s. Samples were analyzed on a mass spectrometer (MS; Waters Acquity H-Plus ultra-performance liquid chromatography [UPLC] with a Water Xevo TQ-S Cronos, Milford, MA, USA), operated in negative electrospray ionization (ESI), utilizing multiple reaction monitoring (MRM) mode. Separation of analytes was obtained using a gradient program on a UPLC column (Waters Acquity BEH C18; 2.1 mm × 150 mm × 1.7 μm) with a mobile phase consisting of water and acetonitrile (both with 0.1% formic acid). The MS was optimized using authentic standards, and samples were quantified against linear calibration curves (ranging from 1 to 2000 μM) using quantitation ion transitions for all the analytes and their corresponding internal standards. Quality control consisted of certified reference material (where available), blanks, duplicates, and spiked samples to assess the precision and accuracy of the analytical method.

Concentrations of 5-hydroxytryptophan (5-HTP), serotonin (5-HT), and 5-hydroxyindoleacetic acid (5-HIAA) were measured in plasma. A 50-μL sample of plasma plus 50 μL internal standard mixture (containing the isotopically labeled internal standards of 500 nM 5-HT-d4 hydrochloride (Cayman Chemical Co., Ann Arbor, MI, USA) 2000 nM 5-HTP-d4 (US Biological, Salem, MA, USA) and 1000 nM 5-HIAA-d6 (Cerilliant Corp, Round Rock, TX, USA) in water with 0.1% formic acid) underwent protein precipitation, via addition of 400 μL ice cold methanol. Samples were vortexed, centrifuged at 16,000× *g* for 10 min at 4 °C, evaporated to dryness in a centrivap (Labconco, Kansas City, MO, USA) at 60 °C for 75 min, and reconstituted with 100 μL of water with 0.1% formic acid (initial mobile phase). Samples were analyzed using UPLC (Acquity H-Plus; Waters Corp., Milford, MA, USA) coupled with a mass spectrometer (MS; Xevo TQ-S Cronos; Waters Corp.), operated in positive electrospray ionization (ESI), utilizing multiple reaction monitoring (MRM) mode. Separation of analytes was obtained using a gradient program on a UPLC column (2.1 mm × 100 mm × 1.8 μm; Waters Acquity HSS T3) with a mobile phase consisting of water and methanol (both with 0.1% formic acid). The MS was optimized using authentic standards, and samples were quantified against linear calibration curves using quantitation ion transitions for all the analytes and their corresponding internal standards. Quality control consisted of blanks, duplicates, and spiked samples to assess the precision and accuracy of the analysis method.

***Muscle biopsy.*** Longissimus muscle biopsy was carried out following Greene et al. [60]. In brief, the lamb was immobilized in a chute to permit side access. A dose of meloxicam (0.5–1 mg/kg body weight) was given orally to the animal. The back was clipped over the longissimus muscle site at the 10th to 13th rib area for a length of about 8 cm and rinsed with warm water to remove any debris if necessary. The site was prepared for aseptic surgery using triplicate scrubs of 2% chlorhexidine followed by a 70% isopropyl alcohol rinse. After the third application, local anesthetic (lidocaine HCL; 20 mg/mL) was administered subcutaneously in a diamond pattern using four injection sites. The area was sprayed with 5% betadine solution and given 5 min to ensure numbness. An L-shaped incision (1 cm by 1 cm) was made with a sterile scalpel. The longissimus muscle (LM) biopsy was extracted from the 15 lambs (5 lambs per day) using a sterile biopsy punch (8 mm). A target of 0.2 g of muscle tissue was extracted from the animal per biopsy. The site was closed with a surgical staple and monitored daily for seven days to ensure proper healing. Staples were removed after complete healing on day seven.

***Transcriptomics.*** The longissimus muscle sample was extracted and snap-frozen in liquid nitrogen before storing at −80 °C for future RNA extraction. The snap-frozen longissimus muscle samples were crushed and weighed. RNA was extracted using Trizol (Invitrogen, Thermo Fisher Scientific, Waltham, MA, USA) reagent in combination with the Norgen BioTek RNA extraction kit (Norgen Biotek Corporation, Thorold, ON, Canada; Cat 43200) according to the manufacturer’s procedure. The total RNA was quantified using a NanoDrop 1 spectrophotometer (ThermoFisher, Waltham, MA, USA). RNA integrity numbers (RIN) were generated using the High Sensitivity RNA ScreenTape Assay for Tapestation systems using an Agilent 4200 Tapestation Bioanalyzer (Agilent Technologies, Santa Clara, CA, USA) at the Clemson University Genomics and Bioinformatics Laboratory according to the manufacturer, and all RIN values were above 7. Total RNA samples were stored at −80 °C and later shipped on dry ice to Novogen (Durham, NC, USA) for library preparation and sequencing. A minimum of 100 ng/µL of total RNA was required for library preparation. The total RNA concentration of our samples was above the requirement; a total of 2 µL of the total RNA was diluted with molecular-grade water in a 2 mL RNase-free tube before shipping, and the remaining RNA was stored at −80 °C for further use. Raw data was sent by Novogene, and general statistics for quality control were carried out using FastQC (https://github.com/s-andrews/fastqc (accessed on 17 March 2025)).

Raw data was cleaned by removing reads containing adapters and low-quality bases. Afterwards, we calculated fastp total reads after filtering, GC content after filtering, percentage of reads passing filter, percentage of adapter-trimmed reads, and FastQC total sequences. Total reads were obtained after filtering and genome assembly for all Ovis aries genomes. ARS_UI_Ramb_v3.0 was used as a reference genome for read alignment (https://www.ncbi.nlm.nih.gov/datasets/genome/GCF_016772045.2/ (accessed on 21 October 2024)). Only mapped genes were further analyzed. After analysis, all sample gene counts were obtained from FeatureCounts, and normalization was carried out using DESeq2 normalization. DESeq analysis was used to obtain differential expression of genes (DEGs). Raw read counts were loaded into RStudio (R4.4.3) from subread all sample gene counts, and different R packages and libraries, which include limma, edgeR, ggplot2, splines, grid, lubridate, and tidyverse, were loaded for analysis. Size factors, model counts, and dispersions were estimated using the DESeq2 pipeline on an everyday binomial distribution basis. Pairwise comparison was carried out between each treatment group (5-HTP vs. CON, GAA vs. CON, and GAA vs. 5-HTP). Benjamin–Hochberg was used for adjusted *p* < 0.05, and an absolute log2 fold change > 1 was used to identify differentially expressed genes. Principal Component Analysis was used to visualize the sample clustering using DESeq2’s plotPCA function, while DESeq results were visualized using a heatmap and a volcano plot.

For DEG result validation, mRNA cDNA was obtained from the isolated RNA using qScript cDNA (Quanta, 95048, Beverly, MA, USA) Supermix according to the manufacturer. The mRNA cDNA was used to analyze PAX-7, MYOD, MYC, ENOX2, and MS4A8 using quantitative real-time PCR (qPCR) using PowerUp (Thermo Fisher, Waltham, MA, USA) SYBR Green Master Mix and the QuantStudio 3 Mastercycler (ThermoFisher, Waltham, MA, USA). The PCR program was run in a standard cycling mode, with uracil-DNA glycosylase (UDG) activation at 50 °C and enzyme activation at 95 °C, both for 2 min and 1 cycle. It also went through 95 °C denaturing for 15 s and annealing/extending at 60 °C, both at 40 cycles. The hold also included a ramp rate of 1.6 °C/s to 95 °C for 15 s, 1.6 °C/s to 60 °C for 1 min, and 0.075 °C/s to 95 °C for 15 s. UXT and EIF3K were used as reference genes, and geometric mean calculations were performed. Relative gene expression levels were calculated using the 2^−ΔΔCT^ method [65].

***miRNA.*** According to the manufacturer’s instructions, the TaqMan miRNA RT Kit obtained miRNA cDNA from the isolated RNA. Quantitative real-time RT-PCR (qPCR) using the TaqMan Fast Advanced Master Mix (ThermoFisher) and the Quant Studio3 was used to obtain miRNA cDNA according to the manufacturer’s instructions to analyze miR-133a-3p and miR-133a-5p (ThermoFisher TaqMan MicroRNA Assays). U6 (ThermoFisher TaqMan MicroRNA Assays) was used as a reference gene allowing for accurate comparison by variations in RNA samples. Relative gene expression levels were calculated utilizing the 2^−ΔΔCT^ method [65].

***Statistical analysis.*** For relative gene expression data, treatment differences were determined using Analysis of Variance (ANOVA) using JMP PRO 18.0.2 (785088) and LSMeans pair-comparison Student’s *t*-test. For animal growth and muscle area, the model included treatment, day, and the two-way interaction. Day was significant (*p* < 0.05), and means were separated using a protected least significant difference test. Lamb was the experimental unit. Significance was determined at *p* < 0.05. Power was adequate (*p* = 0.90) with five animals per treatment to detect differentially expressed genes (α = 0.05) based on previous RNA-Seq experiments in ovine skeletal muscle [55].

## 5. Conclusions

In conclusion, our findings indicate that 5-HTP and quercetin supplementation altered SC proliferation, whereas GAA influenced SC differentiation in vitro. In vivo, 5-HTP and GAA supplementation for 28 d did not alter muscle mass, but GAA did alter the transcriptome. According to the pathway enrichment analysis, glycine–serine–threonine metabolism and cysteine–methionine metabolism are the amino acid metabolism pathways with the highest number of pathway genes, indicating that GAA affects amino acid utilization and downstream energy metabolism, which are functions characteristic of a precursor to creatine. 5-HTP supplementation increased 5-HIAA, a breakdown product of serotonin, but did not alter skeletal muscle transcriptome in this study. One limitation of the study is that the concentration of the supplemented compounds in the skeletal muscle tissue was not determined due to the limitation of sample size with the use of a skeletal muscle biopsy. Although 5-HTP, GAA, and quercetin showed evidence of a direct cellular response in vitro, it is unknown if they elicited a direct response in vivo when administered sublingually at these concentrations.

## Figures and Tables

**Table 1:** Liquid chromatography–mass spectrometry analysis of plasma metabolites in lambs supplemented via sublingual delivery with 5-hydroxytryptophan (5-HTP) or guanidinoacetic acid (GAA) in lambs for 28 d. ^ab^ Means with uncommon superscripts in the same row differ (*p* < 0.05).

Metabolite, nM	CON	GAA	5-HTP	*p*-Value
5-Hydroxytryptamine (5-HT)	1238.44	942.58	2311.44	>0.05
5-Hydroxytryptophan (5-HTP)	5.30	5.00	5.36	>0.05
5-Hydroxyindoleacetic acid (5-HIAA)	102.22 ^b^	100.92 ^b^	225.55 ^a^	<0.01
Acetate	557.16	471.99	469.75	>0.05
Hydroxybutyrate	452.15	514.11	499.31	>0.05
Propionate	13.73	11.99	14.13	>0.05
Isobutyrate	0.98	1.22	1.21	>0.05
Butyrate	10.32	11.17	13.26	>0.05
Isovalerate + 2-Methylbutyrate	1.42	1.60	1.96	>0.05
Hippurate	16.63	14.57	14.02	>0.05
Succinate	8.21	8.18	6.38	>0.05
Guanidinoacetic acid (GAA)	2.03	5.42	2.93	>0.05

**Table 2:** Guanidinoacetic acid (GAA) enriched pathways with responsive genes. Pathways with FGR < 0.05 are considered significant.

Enrichment FDR	nGenes	Pathway Genes	Fold Enrichment	Pathway
0.001458291	1	4	2742.75	Path: oas00290 Valine, leucine and isoleucine biosynthesis
3.29E−05	2	45	487.6	Path: oas00260 Glycine, serine and threonine metabolism
0.0089050655	1	44	249.3409091	Path: oas00860 Porphyrin metabolism
0.008020655	1	49	223.8979592	Path: pas00270 Cysteine and methionine metabolism
0.008978446	1	74	148.2567568	Path: oas01230 Biosynthesis of amino acids
0.012052138	1	116	94.57775862	Path: oas01200 Carbon metabolism
0.013263838	1	146	75.14383562	Path: oas01240 Biosynthesis of cofactors
0.0058020655	2	1554	14.11969112	Path: oas01100 Metabolic pathways

## Data Availability

The RNA-Seq dataset presented in this study can be found in online repositories for NCBI, BioProject: PRJNA1489786, Effects of amino acid derivatives on skeletal muscle transcription.

## Supplementary Materials

The following supporting information can be downloaded at: https://www.mdpi.com/article/10.3390/muscles5030052/s1, Table S1: General statistics for filtered sequencing data from mRNA Sequencing for Control, 5-hydroxytryptophan (5-HTP) and guanidinoacetic acid (GAA). Figure S1: Representative images of EDU-stained SC with 5-hydroxytryptophan (5-HTP), guanidinoacetic acid (GAA) or quercetin (QUE) supplementation at different concentrations (0, 12.5, 25 or 50 umol/L). Figure S2: Representative images of MYOG-stained SC with 5-hydroxytryptophan (5-HTP), guanidinoacetic acid (GAA) or quercetin (QUE) supplementation at different concentrations (0, 12.5, 25 or 50 umol/L).

## Abbreviations

The following abbreviations are used in this manuscript:

GAA	Guanidinoacetic acid
5-HTP	5-Hydroxytryptophan
5-HT	5-Hydroxytryptamine (serotonin)
5-HIAA	5-hydroxyindoleacetic acid
SC	Satellite cells
miRNA	microRNA
LC-MS	Liquid chromatography–mass spectrometry
EDU	5-ethynyl-2′-deoxyuridine
Ct	Cycle threshold
LMA	Longissimus muscle area
FDR	False discovery rate
qPCR	Quantitative polymerase chain reaction
