# Supplementary material for: Effect of Amino Acid Derivatives and Polyphenol Supplementation on Ovine Muscle Growth In Vitro and In Vivo
Source: Muscles. 2026 Jul 17;5(3):52. doi: 10.3390/muscles5030052 (PMC13397929; doi:10.3390/muscles5030052)
Supplement: Supplementary file 1 [file muscles-05-00052-s001.zip › muscles-4398216-supplementary.pdf]

Supplementary Materials

Table S1. General statistics for filtered sequencing data from mRNA Sequencing for Control, 5-hydroxytryptophan (5-HTP) and guanidinoacetic acid (GAA).

Sample name	Reads after filtering (M)	% GC content	% reads passing filtering	% Adapter trimmed read	% >Q30
Control	66.6	51.0	99.5	4.6	97.8
Control	60.7	51.3	99.5	4.9	97.8
Control	53.9	52.2	99.4	5.8	97.8
Control	66.5	51.1	99.4	7.0	97.7
GAA	59.5	51.3	99.5	4.9	97.7
GAA	51.8	51.4	99.0	4.6	97.4
GAA	65.7	52.4	99.5	4.7	97.8
GAA	67.2	51.3	99.4	4.7	97.6
5-HTP	56.5	51.0	99.5	5.2	97.8
5-HTP	50.0	50.8	99.5	4.2	97.8
5-HTP	65.5	50.7	99.5	4.8	97.9
5-HTP	67.4	51.0	99.4	4.3	97.8

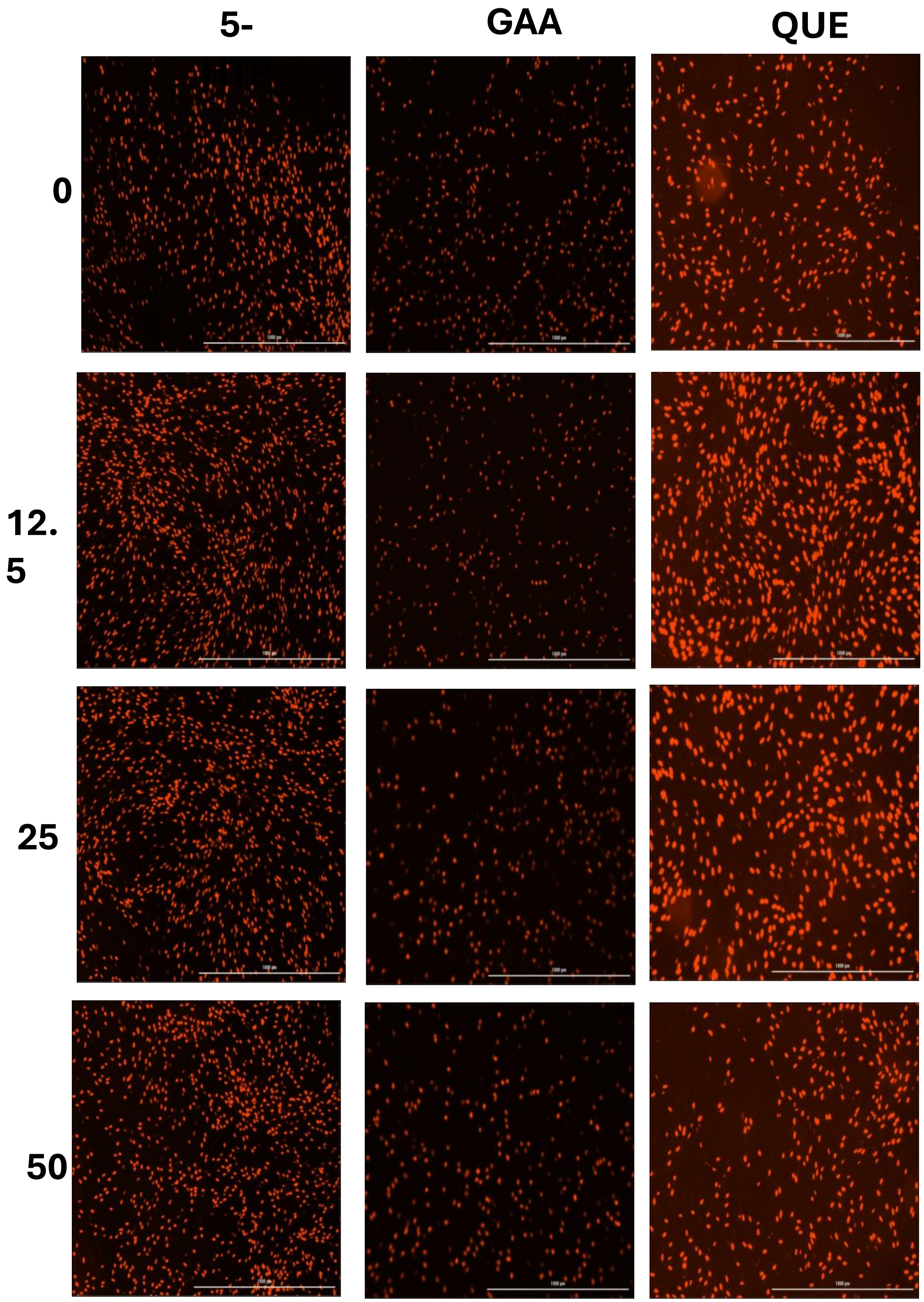

Figure S1. Representative images of EDU-stained SC with 5-hydroxytryptophan (5-HTP), guanidinoacetic acid (GAA) or quercetin (QUE) supplementation at different concentrations (0, 12.5, 25 or 50 umol/L).

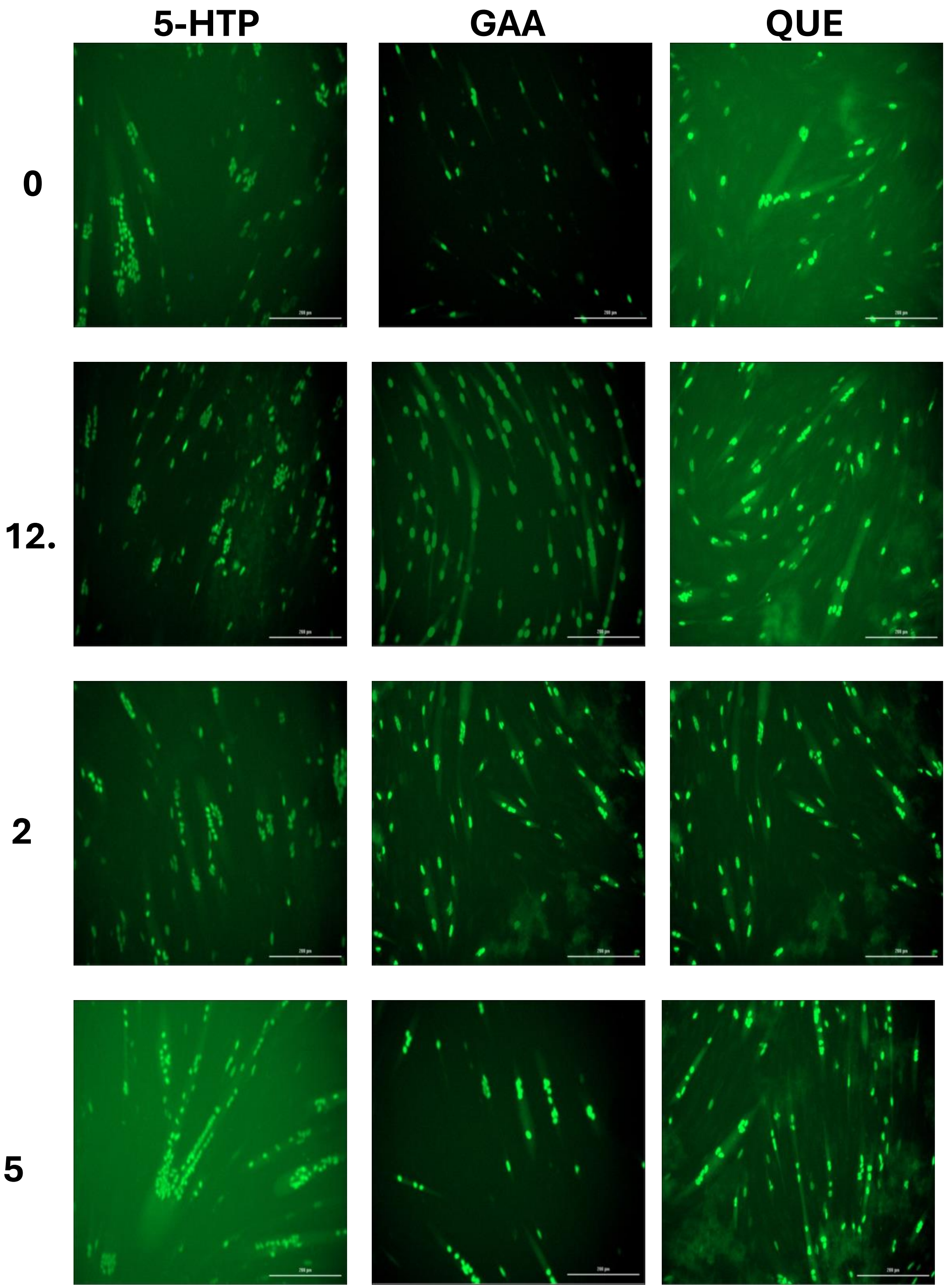

Figure S2. Representative images of MYOG-stained SC with 5-hydroxytryptophan (5-HTP), guanidinoacetic acid (GAA) or quercetin(QUE) supplementation at different concentrations (0, 12.5, 25 or 50 umol/L).
